# Circovirus Hepatitis in Immunocompromised Patient, Switzerland

**DOI:** 10.3201/eid3010.240678

**Published:** 2024-10

**Authors:** Baptiste Hamelin, Philippe Pérot, Ian Pichler, Jasmin D. Haslbauer, David Hardy, David Hing, Sarra Loulizi, Béatrice Regnault, Anouk Pieters, Ingmar Heijnen, Caroline Berkemeier, Maria Mancuso, Verena Kufner, Niels Willi, Anne Jamet, Nolwenn Dheilly, Marc Eloit, Mike Recher, Michael Huber, Kirsten D. Mertz

**Affiliations:** Cantonal Hospital Baselland, Liestal, Switzerland (B. Hamelin, M. Mancuso, N. Willi, K.D. Mertz);; University Hospital Basel, Basel, Switzerland (B. Hamelin, J.D. Haslbauer, I. Heijnen, C. Berkemeier, M. Mancuso, N. Willi, M. Recher, K.D. Mertz);; Institut Pasteur, Paris, France (P. Pérot, D. Hardy, D. Hing, S. Loulizi, B. Regnault, N. Dheilly, M. Eloit);; University of Zurich, Zurich, Switzerland (I. Pichler, V. Kufner, M. Huber);; University of Basel Department of Biomedicine, Basel (A. Pieters, M. Recher, K.D. Mertz);; Assistance Publique–Hôpitaux de Paris Centre Université de Paris Cité Necker-Enfants Malades Hospital, Paris (A. Jamet)

**Keywords:** circovirus, porcine circovirus, hepatitis, immunosuppression, transmission, viruses, Switzerland

## Abstract

We identified a novel human circovirus in an immunocompromised 66-year-old woman with sudden onset of self-limiting hepatitis. We detected human circovirus 1 (HCirV-1) transcripts in hepatocytes and the HCirV-1 genome long-term in the patient’s blood, stool, and urine. HCirV-1 is an emerging human pathogen that persists in susceptible patients.

Circoviruses are an emerging group of DNA viruses with largely unknown pathogenicity in humans (*1*,*2*). The best-studied circovirus is porcine circovirus 2, which causes hepatitis in pigs, among other diseases (*3*,*4*). Novel human circovirus 1 (HCirV-1) was recently linked to chronic infection and liver damage in an immunosuppressed patient (*5*).

## The Study

**Figure 1:**
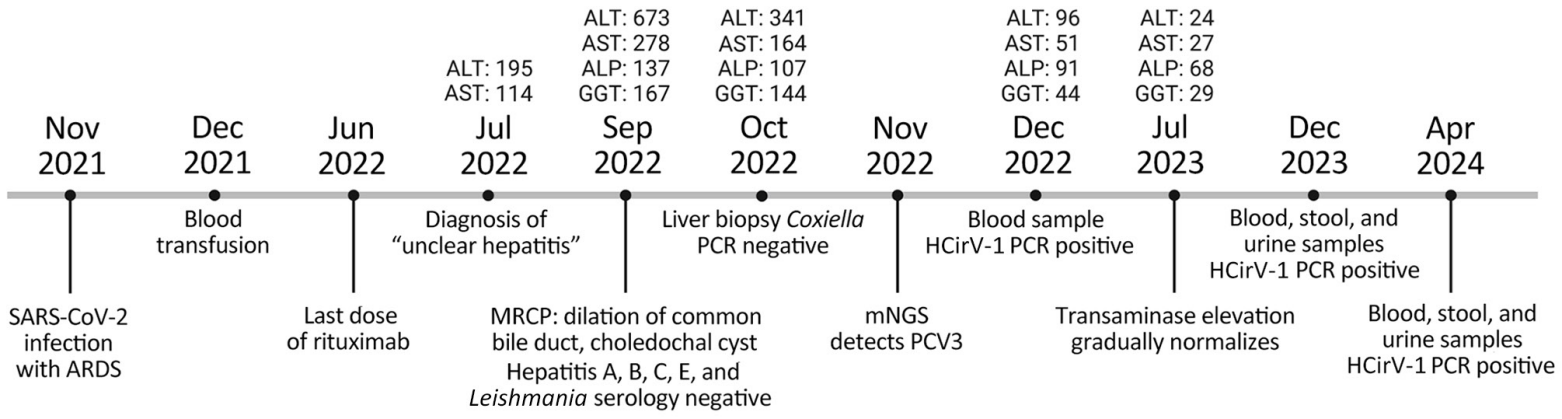
Clinical course of patient with hepatitis of unknown origin, Switzerland, November 2021–April 2024. Top of timeline shows results of enzyme level testing, expressed as units per liter; bottom of timeline shows clinical course. ALP, alkaline phosphatase; ALT, alanine aminotransferase; ARDS, acute respiratory distress syndrome; AST, aspartate aminotransferase; GGT, gammaglutamyl transferase; HCirV-1, human circovirus; mNGS, metagenomic next-generation sequencing; MRCP, magnetic resonance cholangiopancreatography; PCV3, porcine circovirus 3.

A 66-year-old woman who sought care at a regional hospital in Switzerland had sudden elevation of hepatic transaminases in July 2022. Transaminases reached peak values in September 2022, without concurrent elevation of autoantibody in serologic tests (Figure 1). Results of serologic tests for hepatitis viruses A, B, C, and E were negative. When cytolytic hepatitis was diagnosed, the patient had had rheumatoid arthritis for 20 years, which had been treated with a daily dose of prednisolone (5 mg), intravenous rituximab (1,000 mg at 6-month intervals), and intermittent methotrexate. The most recent rituximab was given in June 2022, and methotrexate had been paused. Eight months before onset of hepatitis, the patient had been hospitalized for 2 months with SARS-CoV-2–associated acute respiratory distress syndrome (November–December 2021). During that hospitalization, she had a small intestine diverticular perforation, which required surgery, and macrocytic anemia, for which she received a blood transfusion. Throughout hospitalization, she received additional corticosteroids.

The patient lives alone in rural Switzerland, without direct contact with animals. She had not traveled outside Switzerland during the past 8 years. Her dietary habits were unremarkable except for sporadic consumption of raw calf liver, which she most recently consumed before 2018. She eats cured pork and beef products and gets milk from the supermarket.

Because of the unclear increase in liver enzymes, the treating physician performed a liver biopsy in October 2022 (Figure 1). Histologic analysis revealed acute and subacute hepatitis with a periportal mixed inflammatory infiltrate consisting of lymphocytes, histiocytes, plasma cells, and neutrophilic and eosinophilic granulocytes (Appendix Figure 1). Necroinflammatory foci associated with fat droplets, fibrin, or both were reported. The pathologist categorized the changes most likely as infectious hepatitis. Histologic differential diagnoses included drug-related hepatitis and autoimmune hepatitis, both of which seemed highly unlikely (i.e., no change in medication and no autoantibodies in serologic tests). Several pathogens, including *Coxiella burnetii* bacteria, cytomegalovirus, Epstein-Barr virus, and *Leishmania*, were ruled out by laboratory testing ordered by the treating physician. Transaminase levels gradually decreased and eventually normalized in July 2023.

To clarify the origin of the hepatitis, we analyzed the liver biopsy with a metagenomic next-generation sequencing workflow to identify pathogens (*6*–*8*). We identified some reads that were initially assigned to porcine circovirus 3 (PCV3) (Appendix Table 1). After publication of the HCirV-1 genome (HCirV-1-FR), we incorporated the HCirV-1 genome sequence into our taxonomic profiling index, reanalyzed our sequencing data, and found greater sequence identity with HCirV-1 than with PCV3 or any other circovirus (Appendix Table 2). We analyzed sequencing data by using Microseek to help identify more distant sequences (*9*). We deposited the full-length genome sequence of the HCirV-1 strain from Switzerland (HCirV-1-CH) into GenBank (accession no. OR905605).

**Figure 2:**
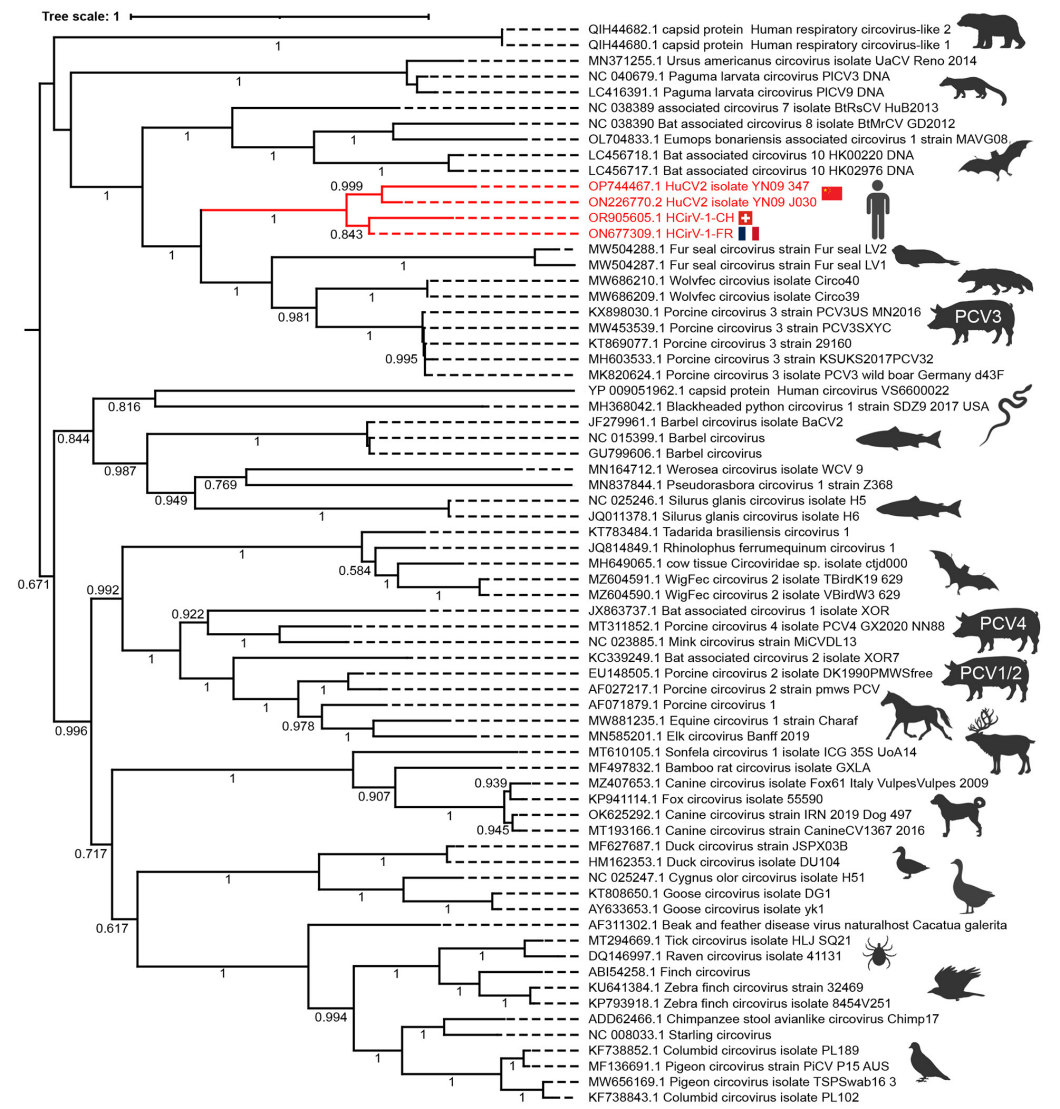
Phylogenetic analysis of the circovirus isolate from a patient with hepatitis of unknown origin, Switzerland (HCirV-1-CH). Capsid protein sequences representative of human and animal circovirus strains are shown. We aligned sequences by using MAFFT (https://mafft.cbrc.jp/alignment/software) under the L-INS-I parameter and performed maximum-likelihood phylogenetic reconstruction through the IQ-Tree portal (http://www.iqtree.org). Red indicates HCirV-1-FR, HCirV-1-CH, and HuCV2 sequences. Tree scale indicates the number of amino acid substitutions per site. Display range for bootstraps is 0.5–1.0. GenBank accession numbers are provided. HCirV-1-CH, human circovirus 1 from patient in Switzerland; HCirV-1-FR, published human circovirus 1 genome from France; HuCV2, human circovirus sequence detected in injection drug users in China (*10*); PCV, porcine circovirus.

The nucleotide identities of HCirV-1-FR and HCirV-1-CH at the full-genome level were 83.6% similar, higher for the polymerase gene (91.2% nucleotide identity, 95.6% amino acid identity) and lower for the capsid gene (69.4% nucleotide identity; 64.1% amino acid identity). Phylogenetic analysis based on the capsid protein clustered HCirV-1-FR and HCirV-1-CH together and indicated that our patient had a novel strain of HCirV-1. HCirV-1-FR and HCirV-1-CH cluster closely with the circovirus sequence recently described in drug users in China (*10*). HCirV-1-FR, HCirV-1-CH, and the strains from China form a new phylogenetic clade distinct from other animal circoviruses (Figure 2).

**Figure 3:**
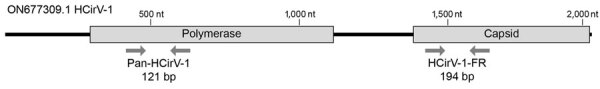
Nonhuman metagenomic next-generation sequencing reads from liver biopsy of hepatitis patient in Switzerland mapped against the human circovirus genome (Appendix Tables 1, 2). Positions of the putative sequences for polymerase and capsid proteins and the 2 primer pairs (HCirV-1-FR, Pan-HCirV-1) used for the PCRs are indicated. HCirV-1-FR, published human circovirus 1 genome from France (GenBank accession no. ON677309.1); Pan-HCirV-1, primers adapted to target the broadening clade of human circoviruses.

**Figure 4:**
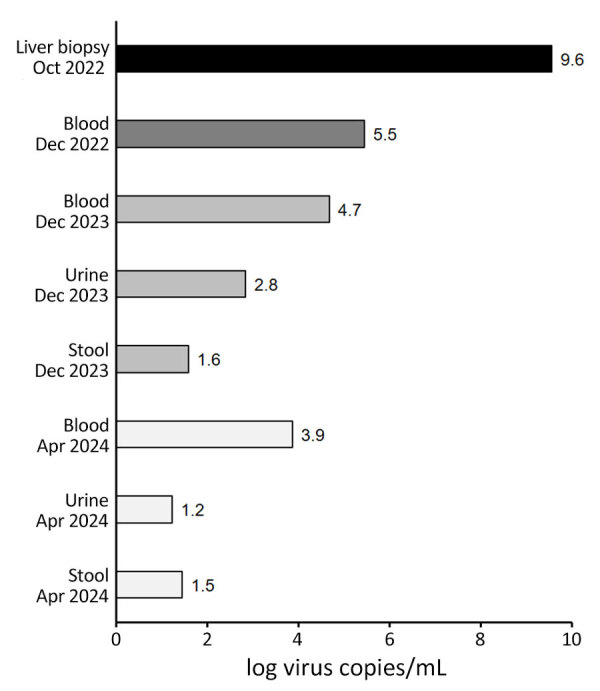
Human circovirus 1 viral loads in recently collected and archival samples (2023 and 2024) compared with viral load in initial liver biopsy (2022) from a hepatitis patient, Switzerland. Virus copies and log virus copies were also tabulated (Appendix Table 3).

We confirmed the presence of HCirV-1 in the liver biopsy by using HCirV-1–specific PCRs. We used HCirV-1-FR–specific primers and a second pair of primers adapted to target the broadening clade of human circoviruses, including the strains from China, but not any animal strains (Figure 3; Appendix Figure 2) (*5*,*11*). Although both primer sets successfully amplified the HCirV-1-CH strain despite several mismatches between HCirV-1-FR primers and HCirV-1-CH, the second pair of primers led to more efficient amplification, so we used them for subsequent quantification. We found HCirV-1 viral load to be high in the liver biopsy (3.39 × 10^9^ genome copies/g or 3.63 × 10^9^ genome copies/mL) (Figure 4; Appendix Table 3).

Archival formalin-fixed paraffin-embedded tissues of the patient were available, including biopsies from the gastrointestinal tract taken before the diagnosis of circovirus hepatitis. We tested all of those tissue samples with HCirV-1–specific PCRs, and the results were negative (i.e., we did not detect HCirV-1 before hepatitis nor outside the liver) (Appendix Table 4).

We retrospectively analyzed an archival blood sample from the period when the patient had hepatitis (December 2022) by using HCirV-1–specific PCRs; results were positive, indicating viremia (Figure 4; Appendix Table 3). In more recent blood, stool, and urine samples (taken December 2023–April 2024 [i.e., 17–21 months after the hepatitis diagnosis]), we still detected HCirV-1 in blood; viral loads remained high. We detected HCirV-1, albeit with lower viral loads, in urine and stool (Figure 4; Appendix Table 3). We also detected HCirV-1 in a saliva sample (April 2024), albeit with a low viral load. Those data confirm HCirV-1 persistence in this patient for >21 months and that the patient continuously shed the virus. We re-sequenced HCirV-1-CH in the most recently collected blood and could not detect notable changes in the genome, meaning that the virus has not mutated over time, which was consistent with low selection pressure from a suppressed immune system. Because the patient had been transfused with an erythrocyte concentrate (December 2021), we analyzed the donor’s serum by using HCirV-1–specific PCRs, which were negative, showing that the virus had not been acquired during blood transfusion.

To characterize the tropism of HCirV-1-CH, we used RNAscope in situ hybridization (Bio-Techne, https://www.bio-techne.com) to localize the HCirV-1-CH nucleic acids in the patient’s liver tissue. The probes targeting HCirV-1-CH stained ≈40% of the hepatocytes (Appendix Figure 3), whereas no staining of normal liver tissues (n = 3) or of liver tissues from patients infected with hepatitis viruses B or C (n = 4) occurred (Appendix Table 5). The areas with the strongest positivity co-localize with the nuclei, which is consistent with nuclear replication of circoviruses and therefore supports the assumption of replicative activity of the virus, even if the RNA cannot be quantified.

This patient had a moderately severe antibody deficiency and missing B cells consistent with years of immunosuppressive therapy (Appendix Table 6). She also had a dysfunction in the activation of the complement system through the mannose-binding lectin pathway. Low numbers of B cells and immunoglobulins probably contributed to the persistence of HCirV-1-CH in this patient.

## Conclusions

We report a case of HCirV-1–associated hepatitis (*5*) that strengthens the assumption that circoviruses are emerging new pathogens in humans, particularly among immunosuppressed patients (*5*,*10*). Our study provides clues to the understanding of HCirV-1 pathobiology and transmission. We ruled out human-to-human transmission through blood transfusion. We show that the virus can persist in humans for a prolonged period and that it is shed in body fluids, thus posing a risk for horizontal transmission in human populations. Of note, parallels can be drawn with porcine circoviruses, which also have an exceptional ability to cause persistent infections and to be shed in fluids, enabling them to spread rapidly in pig populations worldwide after their initial emergence (*12*). In tandem with testing more donor plasma pools, wastewater testing for HCirV-1 could elucidate the regional distribution and prevalence of HCirV-1 infections.

Appendix Additional information about circovirus hepatitis in an immunocompromised patient, Switzerland.
