# Supplementary material for: Circovirus Hepatitis in Immunocompromised Patient, Switzerland
Source: Emerg Infect Dis. 2024 Oct;30(10):2140–4. doi: 10.3201/eid3010.240678 (PMC11431922; doi:10.3201/eid3010.240678)
Supplement: Appendix — Additional information about circovirus hepatitis in an immunocompromised patient, Switzerland. [file 24-0678-Techapp-s1.pdf]

Article DOI: https://doi.org/10.3201/eid3010.240678

EID cannot ensure accessibility for supplementary materials supplied by authors. Readers who have difficulty accessing supplementary content should contact the authors for assistance.

Circovirus Hepatitis in an Immunocompromised Patient, Switzerland

Appendix

Supplementary Results

Immunophenotyping of the patient

The patient has been immunosuppressed for years due to rheumatoid arthritis. During the COVID-19 pandemic (11/2021), the patient had a severe SARS-CoV-2 infection with ARDS, which resulted in weeks of hospitalization and suggested a reduced immune status. We therefore wanted to take a closer look at the patient's immunological profile. We found that the patient had a moderately severe antibody deficiency (IgG 4.6g/l) (Appendix Table 6). All IgG subclasses except IgG2 were reduced. IgM and IgA in the serum were normal. This constellation is typical, although not specific for secondary immunodeficiencies but, like missing B cells, is also consistent with chronic anti-CD20 therapy (rituximab). The CD4 and CD8 T cell counts of the T cells were normal. Among the CD4 T cell subsets, the low frequency of follicular T helper cells are particularly conspicuous, yet again consistent with chronic B cell depletion.

The patient also has a dysfunction in the activation of the complement system via the mannose binding lectin (MBL) pathway (Appendix Table 6). This dysfunction is usually due to genetic variations or mutations in the MBL gene leading to lower levels of MBL protein or dysfunctional MBL that cannot bind properly to pathogen surfaces (*1*–*3*). It occurs relatively frequently in the population (approx. 5%). The functional disruption of the MBL pathway impairs a critical component of the immune response and, in combination with immunosuppressive medication, could increase susceptibility to a variety of infectious diseases including viral infections (*4*–*6*). Altogether, the patient's reduced immune status is consistent with years of anti-CD20 therapy. Low numbers of B cells and immunoglobulins likely contribute to the persistence of HCirV-1-CH in this patient.

Materials and Methods

Ethics statement

This study was conducted according to the principles of the Declaration of Helsinki. The study protocol was reviewed and approved by the Ethics Committee of Northwestern and Central Switzerland (Project-ID 2023–02250). Patient consent was obtained for sample collection and research use.

The patient was included in the prospective immune dysregulation cohort as approved by the local review board (Ethics Committee of Northwestern and Central Switzerland, project-ID 2015–187).

Nucleic acid extraction

Formalin fixed, paraffin embedded (FFPE) blocks were sectioned and incubated with Proteinase K overnight. DNA extraction was performed on an EZ2 Connect automate (Qiagen, Hilden, Germany) with the EZ1&2 DNA Tissue kit (Qiagen, 953034). The other clinical samples were extracted on the EMAG system (bioMérieux, Craponne, France), according to the manufacturer's instructions.

PCR

Polymerase chain reaction was performed with the GeneAmp Fast PCR Master Mix (Thermo Fisher Scientific, Waltham, MA, USA, 4359187). The primers designed to investigate the original HCirV-1 case from Paris (HCirV1-Fw1 5′-ACCTGGATGGACCCTGGAAT-3′ and HCirV1-Rv1 5′-AGAGTTCCACCAGGTTCTGC-3′) were used (*7*). A second set of primers (HCirV-Fw1 5′-TTAGAAGCGGCCAAGGGGA-3′ and HCirV-Rv1 5′-CGCTGCTGTCTCCAAATCCA-3′) was designed to target human circoviruses only (*8*). PCR was performed for 35 cycles with an annealing temperature of 58°C. The PCR product was analyzed via capillary electrophoresis on a Qiaxcell Connect device (Qiagen). DNA extracted from the liver biopsy in which HCirV-1-CH was first identified by mNGS was used as a positive control for all PCRs.

Quantification of viral load

Real-time qPCR was performed using the TaqMan RNA-to-Ct 1-Step Kit on a QuantStudio 7 Flex System (Thermo Fisher Scientific) with samples tested in duplicates as

previously described (*9*). A Pan-HCirV-1-specific probe was designed (5′-FAM-TTACCTGGAGATTGGAGAGAGTGGCTGTGC-TAMRA-3′) and validated before qPCR analysis. A positive control containing both PCR targets, flanked on either side by 50 bases, was serially diluted and quantified by digital PCR (dPCR) to generate a standard curve. dPCR was performed using the QIAcuity OneStep Advanced Probe Kit (Qiagen). Linear regression was performed by plotting the cycle threshold (Ct) values against the log-transformed dPCR concentrations to calculate the absolute quantities of the viral load in the clinical samples.

Metagenomic sequencing and pathogen detection

DNA (250ng) extracted from the liver biopsy was used to generate a whole genome sequencing library with the Ion Plus Fragment Library kit (Thermo Fisher Scientific, 4471252). The library was quantified with the Ion Library TaqMan Quantification kit (Thermo Fisher Scientific, 4468802) and sequenced on an Ion Torrent GeneStudio S5XL (Thermo Fischer Scientific). Taxonomic profiling was performed with the CLC Genomics Workbench software (Qiagen) (*10*). The initial analysis was performed with a viral taxonomic index lacking the HCirV-1 genome (ON677309) (*7*). Because an infection with the novel circovirus was suspected, the taxonomic index was modified and the HCirV-1 genome was included.

For all other samples, library preparation and bioinformatic analysis were performed using the VirMet pipeline (https://github.com/medvir/VirMet/releases/tag/v1.1.1) as previously described (*11*).

Phylogenetic analysis of capsid protein sequences of circoviruses

Sequences were aligned with Multiple Alignment using Fast Fourier Transform (https://www.ebi.ac.uk/Tools/msa/mafft) under the L-INS-I parameter, and maximum-likelihood phylogenetic reconstruction was performed with PhyML implemented through the IQ-TREE portal (http://www.iqtree.org/). GenBank accession numbers for reference sequences and graphic representations of the animal of origin are shown. Scale bar = number of amino acid substitutions per site.

Duplex RNAscope in situ hybridization

Antisense probes targeting both the capsid and the Rep gene (20ZZ probes in total) were developed and manufactured by Bio-Techne (https://www.bio-techne.com/; Minneapolis, Minnesota, USA). 5µm sections of FFPE liver tissues from the HCirV-1-CH patient and control

patients (Appendix Table 5) were mounted on positively charged SuperFrost plus slides (Fischer Scientific). The RNAscope ISH assay was performed using an RNAscope 2.5HD Duplex Assay Kit (ACD Bio-Techne, Newark, CA, USA, 32250). Details of the optimized protocol can be found here: https://acdbio.com/rnascope-25-hd-duplex-assay. Slides were incubated with different RNAscope 2-plex probes: Negative control probe (#320751), positive control probe (#313901, HS-PPIB-C1 [green]) and target circovirus RNA probe (V-HCirV-1-C2 [Red]). After 10 hybridization amplification steps and detection into the HybEZ oven, slides were counterstained with 50% hematoxylin solution. Slides were evaluated using a ZEISS AxioScan 7 digital slide scanner and Zen software (3.9.101; Zeiss, Oberkochen, Germany).

Immunophenotyping and serum complement activity assays

Serum immunoglobulin levels, lymphocyte subpopulations and specific complement pathways were assessed as previously described (*12*,*13*).

References

1. Jack DL, Klein NJ, Turner MW. Mannose-binding lectin: targeting the microbial world for complement attack and opsonophagocytosis. Immunol Rev. 2001;180:86–99. PubMed https://doi.org/10.1034/j.1600-065X.2001.1800108.x

2. Eisen DP, Minchinton RM. Impact of mannose-binding lectin on susceptibility to infectious diseases. Clin Infect Dis. 2003;37:1496–505. PubMed https://doi.org/10.1086/379324

3. Ballegaard V, Haugaard AK, Garred P, Nielsen SD, Munthe-Fog L. The lectin pathway of complement: advantage or disadvantage in HIV pathogenesis? Clin Immunol. 2014;154:13–25. PubMed https://doi.org/10.1016/j.clim.2014.06.002

4. Chang WC, White MR, Moyo P, McClear S, Thiel S, Hartshorn KL, et al. Lack of the pattern recognition molecule mannose-binding lectin increases susceptibility to influenza A virus infection. BMC Immunol. 2010;11:64. PubMed https://doi.org/10.1186/1471-2172-11-64

5. Halla MC, do Carmo RF, Silva Vasconcelos LR, Pereira LB, Moura P, de Siqueira ER, et al. Association of hepatitis C virus infection and liver fibrosis severity with the variants alleles of *MBL2* gene in a Brazilian population. Hum Immunol. 2010;71:883–7. PubMed https://doi.org/10.1016/j.humimm.2010.05.021

6. Yilmaz D, Soyoz M, Sahin A, Cerci-Alkac B, Karahan-Coven HI, Ekemen-Keles Y, et al. Association between mannose binding lectin gene polymorphisms and clinical severity of COVID-19 in children. Mol Biol Rep. 2023;50:5871–7. PubMed https://doi.org/10.1007/s11033-023-08524-z

7. Pérot P, Fourgeaud J, Rouzaud C, Regnault B, Da Rocha N, Fontaine H, et al. Circovirus hepatitis infection in heart-lung transplant patient, France. Emerg Infect Dis. 2023;29:286–93. PubMed https://doi.org/10.3201/eid2902.221468

8. Pérot P, Da Rocha N, Farcet MR, Kreil TR, Eloit M. Human circovirus is not detected in plasma pools for fractionation. Transfusion. 2024;64:16–8. PubMed https://doi.org/10.1111/trf.17604

9. Lewandowska DW, Zagordi O, Geissberger FD, Kufner V, Schmutz S, Böni J, et al. Optimization and validation of sample preparation for metagenomic sequencing of viruses in clinical samples. Microbiome. 2017;5:94. PubMed https://doi.org/10.1186/s40168-017-0317-z

10. Nienhold R, Mensah N, Frank A, Graber A, Koike J, Schwab N, et al. Unbiased screen for pathogens in human paraffin-embedded tissue samples by whole genome sequencing and metagenomics. Front Cell Infect Microbiol. 2022;12:968135. PubMed https://doi.org/10.3389/fcimb.2022.968135

11. Kufner V, Plate A, Schmutz S, Braun DL, Günthard HF, Capaul R, et al. Two years of viral metagenomics in a tertiary diagnostics unit: evaluation of the first 105 cases. Genes (Basel). 2019;10:661. PubMed https://doi.org/10.3390/genes10090661

12. Seitz L, Gaitan D, Berkemeier CM, Berger CT, Recher M. Cluster analysis of flowcytometric immunophenotyping with extended T cell subsets in suspected immunodeficiency. Immun Inflamm Dis. 2023;11:e1106. PubMed https://doi.org/10.1002/iid3.1106

13. Meyer BJ, Kunz N, Seki S, Higgins R, Ghosh A, Hupfer R, et al. Immunologic and genetic contributors to CD46-dependent immune dysregulation. J Clin Immunol. 2023;43:1840–56. PubMed https://doi.org/10.1007/s10875-023-01547-y

Appendix Table 1. Initial metagenomic NGS (mNGS) analysis performed on the patient's liver sample*

Species	Abundance	Coverage
PCV3	12	0.99
Xanthomonas phage Xf109	1	0.03
Propionibacterium phage Attacne	1	0.01
Propionibacterium phage PAS50	1	0.01
Propionibacterium virus PHL092M00	1	0.00
Virus (Unknown)	1	0.00
Staphylococcus phage SPbeta-like	1	0.00

*The count table provided by the taxonomic profiling workflow shows a limited number of 12 reads for the porcine circovirus 3 (PCV3) as well as background signals for various phage species. Percent genome coverage is given for each reference.

Appendix Table 2. Reanalysis of the original data with an updated taxonomic profiling index including the HCirV-1 genome.

Species	Abundance	Coverage
HCirV-1	4461	70.9
HCirV-1 (Unknown)	134	2.1
Heunggongvirae (Unknown)	5	0.0
Loebvirae (Unknown)	1	0.0

The results show a high abundance of reads (4595) allocated to HCirV-1, suggesting that the reads allocated to PCV3 in the initial analysis are actually from HCirV-1 and that the two viruses are related. Percent genome coverage is given for each reference.

Appendix Table 3. Quantification of viral loads in HCirV-1-CH positive samples.

Sample Type	Sampling Date	Ct Values	Virus Copies (copies/ml)	Log Virus Copies (copies/ml)
Liver Biopsy	2022 Oct	26.1	3.63E+09	9.6
Blood	2022 Dec	23.33	2.83E+05	5.5
Blood	2023 Dec	27.6	4.77E+04	4.7
Urine	2023 Dec	32.2	6.97E+02	2.8
Stool	2023 Dec	36.5	3.85E+01	1.6
Blood	2024 Apr	28.68	7.67E+03	3.9
Urine	2024 Apr	37.75	1.69E+01	1.2
Stool	2024 Apr	36.99	2.83E+01	1.5
Saliva	2024 Apr	39.87	4.06E+00	0.6

Appendix Table 4. HCirV-1 PCR analysis of various FFPE tissue samples of the patient.

Year	Month	Organ	Localization	Diagnosis	Circovirus PCR
2015	12	Soft tissue	Pulpa	Vascular malformation	Negative
2016	4	Duodenum	Duodenum	Normal small intestinal mucosa	Negative
2016	4	Esophagus	Esophagus	Normal mucosa	Negative
2016	4	Stomach	Stomach	Normal gastric mucosa	Negative
2018	2	Colon	Colon	Normal large intestinal mucosa	Negative
2018	2	Duodenum	Duodenum	Normal small intestinal mucosa	Negative
2018	4	Kidney	Kidney	Normal kidney and ccRCC	Negative
2019	4	Colon	Colon	Normal large intestinal mucosa	Negative
2019	4	Small bowel	Ileum	Normal small intestinal mucosa	Negative
2021	11	Small bowel	Small bowel	Diverticulitis with serosa perforation	Negative
2021	11	Small bowel	Small bowel	Diverticulitis with serosa perforation	Negative
2023	3	Skin	Face	Sebaceous gland adenoma	Negative
2023	3	Skin	Face	Sebaceous gland adenoma	Negative
2023	7	Stomach	Stomach	Normal gastric mucosa	Negative

Appendix Table 5. Liver tissues for in situ hybridization.

Patient sample	Sex	Age , y	Organ	Diagnosis	Function
A	F	65	Liver	Acute and chronic hepatitis due to HCirV-1-CH	Circovirus hepatitis
B	F	28	Liver	Normal liver	Negative control
C1	M	42	Liver	Normal liver	Negative control
C2	M	42	Liver	Normal liver	Negative control
D1	F	49	Liver	Liver cirrhosis, moderate portal inflammatory activity with mild interphase activity, known hepatitis B and C infection	Negative control
D2	F	49	Liver	Liver cirrhosis, moderate portal inflammatory activity with mild interphase activity, known hepatitis B and C infection	Negative control
E1	F	26	Liver	Chronic hepatitis B with minimal activity	Negative control
E2	F	26	Liver	Chronic hepatitis B with minimal activity	Negative control

Appendix Table 6. Immunophenotyping of the patient in April 2024.

Parameters	Normal value	04/2024
IgG	7.0–16.0 g/l	4.5 g/l
IgG1	4.90–11.40 g/l	2.43 g/l
IgG2	1.50–6.40 g/l	1.76 g/l
IgG3	0.20–1.10 g/l	0.14 g/l
IgG4	0.08–1.40 g/l	0.04 g/l
IgA	0.70–4.00 g/l	0.78 g/l
IgM	0.40–2.30 g/l	0.52 g/l
T cells relative	55%–86% (of lymphocytes)	78%
T cells absolute	742–2750/µl	1312/µl
B cells relative	5%–22% (of lymphocytes)	<1%
B cells absolute	80–616/µl	1/µl
NK cells relative	5%–26% (of lymphocytes)	22%
NK cells absolute	84–724/µl	367/µl
CD4 T cells relative	33%–58% (of lymphocytes)	54%
CD4 T cells absolute	404–1612/µl	913/µl
CD8 T cells relative	13%–39% (of lymphocytes)	26%
CD8 T cells absolute	220–1129/µl	436/µl
CD4 naïve relative	15.7%–54.7% of CD4+ T cells	35.8%
CD4 central memory	8%–28.8% of CD4+ T cells	7.7%
CD4 effector memory	16.8%–57.4% of CD4+ T cells	49.2%
CD4 TEMRA	3.6%–23.2% of CD4+ T cells	7.3%
CD4 follicular T helper	6.9%–19.1% of CD4+ T cells	3.1%
CD4 recent thymic emigrants	14.1%–37.2% of naïve CD4+ T cells	23.8%
CD4 Treg	6.1%–11% of CD4+ T cells	5.7%
CD4 activated	4.5%–15.6% of CD4+ T cells	17.3%
CD8 naïve relative	7%–62.5% of CD8+ T cells	13.1%
CD8 central memory	0.6%–4.4% of CD8+ T cells	1.9%
CD8 effector memory	4.3%–64.5% of CD8+ T cells	24.6%
CD8 TEMRA	8.1%–60.5% of CD8+ T cells	60.4%
CD8 activated	5.7%–48.2% of CD8+ T cells	36.6%
Classical complement pathway	69%–129%	125.0%
Alternative complement pathway	30%–113%	81.0%
Mannose binding lectin pathway	10%–125%	<5%

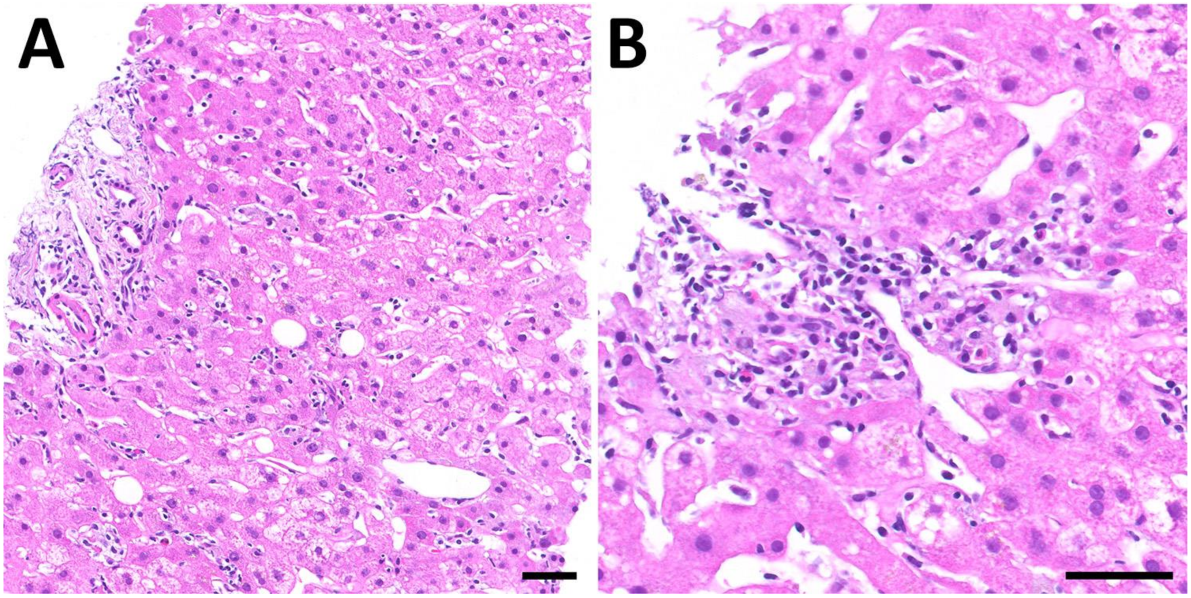

Appendix Figure 1. Hepatitis of unknown origin. H&E staining of the liver biopsy sampled in October 2022. Histology shows acute and subacute hepatitis with a periportal mixed inflammatory infiltrate consisting of lymphocytes, histiocytes, neutrophilic and eosinophilic granulocytes, as well as isolated plasma cells. Scale bars = 50 µm.

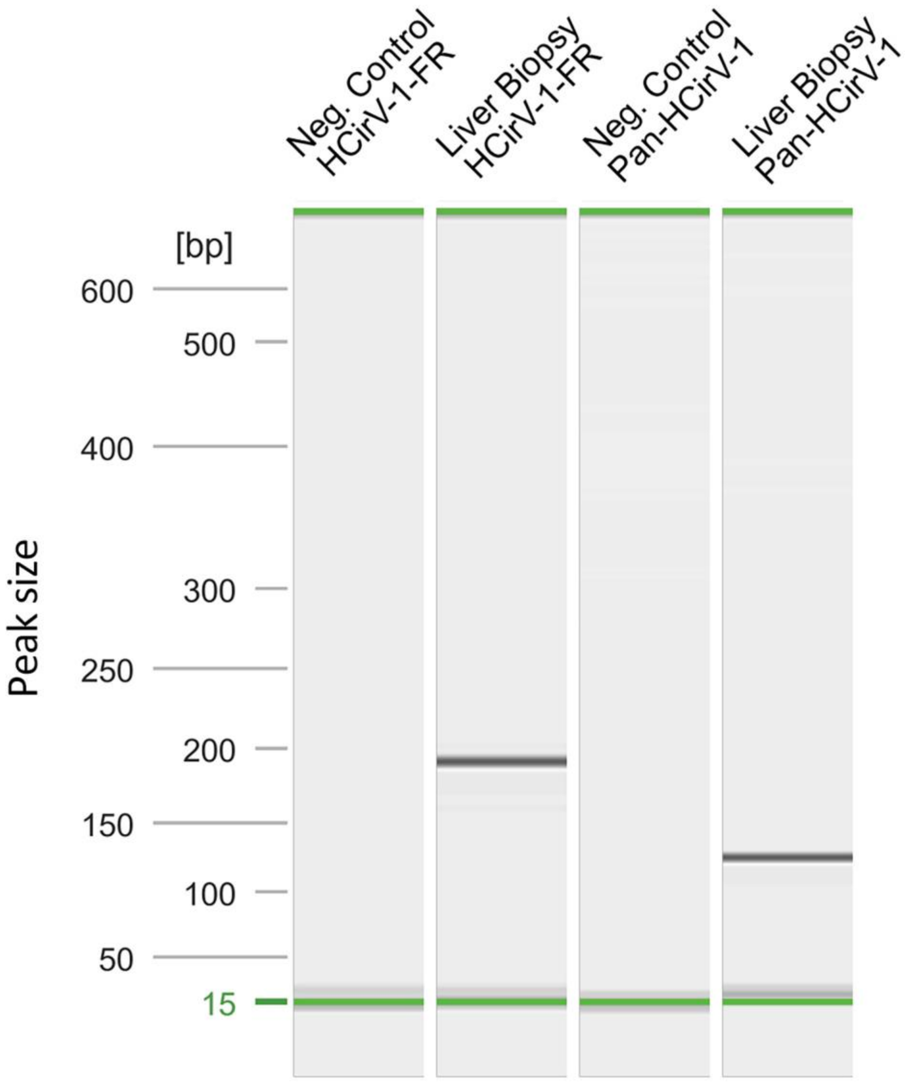

Appendix Figure 2. Reanalysis of our case in the light of the latest publication on circovirus in humans. HCirV-1 specific PCRs performed on DNA obtained from the patient's liver biopsy. HCirV-1-FR specific primers and a second pair of primers (Pan-HCirV-1) adapted to target the broadening clade of human circoviruses including the Chinese strains, but not any animal strains, were used. PCR amplification products are 194bp (HCirV-1-FR) and 121bp (Pan-HCirV-1). The presence of HCirV-1 in the liver biopsy was confirmed.

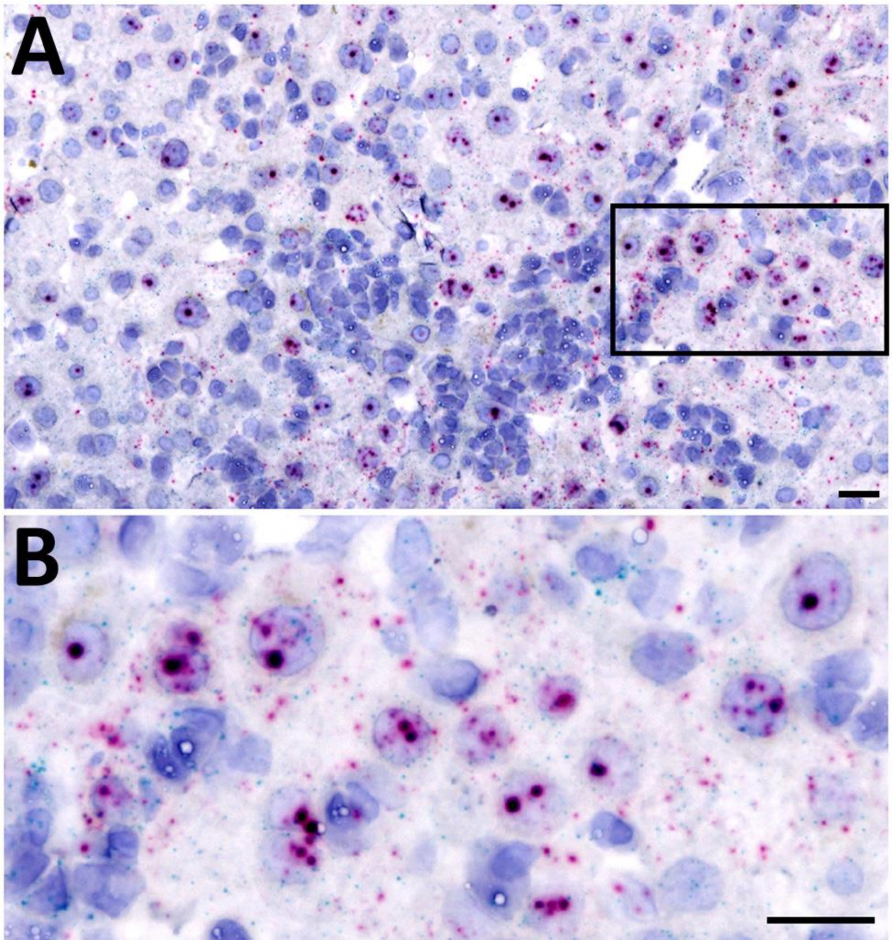

Appendix Figure 3. Liver tropism of HCirV-1. Localization of HCirV-1 nucleic acids (RNA) in the patient's liver tissue using Duplex RNAscope in situ hybridization. *Green*: Positive RNA for the positive control probe HS-PPIB-C1. *Red*: RNAs positive for the circovirus V-HCirV-1-C2 target probe. Nuclei of the liver biopsy are counterstained in hematoxylin (*blue*). Viral nucleic acids can be detected in 40% of hepatocytes. The signals are within the nuclei of the hepatocytes, consistent with nuclear replication of circoviruses. The lower image shows an enlarged section of the upper image. Scale bars = 20µm.
